# Apple Peel Supplementation Potential in Metabolic Syndrome Prevention

**DOI:** 10.3390/life13030753

**Published:** 2023-03-10

**Authors:** Joanna Popiolek-Kalisz, Paweł Glibowski

**Affiliations:** 1 Clinical Dietetics Unit, Department of Bioanalytics, Medical University of Lublin, 20-093 Lublin, Poland; 2 Department of Biotechnology, Microbiology and Human Nutrition, University of Life Sciences in Lublin, 20-704 Lublin, Poland; 3 Department of Cardiology, Cardinal Wyszynski Hospital in Lublin, 20-718 Lublin, Poland

**Keywords:** metabolic syndrome, apple peel, obesity, quercetin, fiber, antioxidants

## Abstract

(1) Introduction: Apples are a source of bioactive substances, e.g., anthocyanidins and flavonols, and dietary fiber. Their highest concentrations are observed in the skin. Metabolic syndrome (MetS) is a set of conditions originally associated with obesity. Excessive adipose tissue accompanying obesity leads to chronic inflammation and metabolic disorders, which result in the development of dyslipidemia, elevated blood pressure, and glucose levels. Thus, supplementation of apple peels, a source of antioxidant substances and fiber, could potentially be a method supporting the prevention of MetS. This paper summarizes the results of available research on the potential impact of apple peel supplementation on the components of MetS. (2) Results: The results from in vitro and animal model studies indicate a positive effect of apple peel supplementation on lipid profile, glucose levels, and blood pressure regulation mediators. Only one human study was performed, and it showed that the consumption of apple peels had an effect on endothelial function but not on other clinical parameters. At the moment, there are no results from observations on large groups of people available. (3) Conclusions: The results of in vitro and animal-model studies indicate the potential of apple peel supplementation in MetS prevention, but it has not been clinically confirmed in human studies. Conducting large human studies could allow a definite clarification of the role of apple peel supplementation in MetS prevention.

## 1. Introduction

Metabolic syndrome (MetS) is a set of disorders that originally are a result of central obesity [1]. Excessive adipose tissue accompanying obesity leads to chronic inflammation and, secondary to that, to atherogenic dyslipidemia, elevated blood pressure, and insulin resistance resulting in elevated glucose levels [1,2,3,4,5]. That is why contracting systemic inflammation by antioxidative substances consumption or supplementation could be potentially a method of MetS prevention. MetS diagnosis requires fulfilling the central obesity criterion, which is defined in Europids as a waist circumference of 94 cm or higher in males or 80 cm or higher in females, plus two out of four additional criteria. They include (I) a triglyceride (TG) level of 150 mg/dL (1.7 mmol/L) or higher or specific treatment for this lipid abnormality; (II) a high-density lipoprotein (HDL) level lower than 40 mg/dL (1.03 mmol/L) in males, or lower than 50 mg/dL (1.29 mmol/L) in females, or specific treatment for this lipid abnormality; (III) a fasting glucose level of 100 mg/dL (5.6 mmol/L) or higher, or previously diagnosed type 2 diabetes; (IV) a systolic blood pressure level of 130 or higher, or diastolic blood pressure level 85 mmHg or higher, or treatment of previously diagnosed hypertension [1]. The previous MetS definition required fulfilling any three out of the mentioned five criteria [1]. It could potentially lead to a situation when a patient with hypertension, diabetes, and dyslipidemia could have been diagnosed with MetS, even though they were not obese. The International Diabetes Federation update underlined the role of central obesity as the foundation of MetS, which is why central obesity is the main criterion that has to be fulfilled for the further assessment and potential diagnosis of MetS. MetS is a serious healthcare problem as it is estimated that 39% men and 32.8% women in Poland have MetS [6]. That is why looking for new methods of MetS prevention and treatment is one of the main targets of public health [7].

Fruit and vegetable consumption is part of WHO dietary recommendations; however, they are not precise in referring to the type or parts of recommended fruit [8]. Moreover, some studies revealed that general fruit consumption does not impact MetS severity, which is why it is important to indicate and test particular products which are beneficial in terms of MetS [9,10].

Apples are one of the most commonly consumed fruits. They are relatively low-caloric (52 kcal/100 g), which can be considered an advantage in terms of MetS dietary prevention. Apples contain low amounts of protein (0.26 g/100 g) or fat (0.17/100 g) and are high in carbohydrates (13.8 g/100 g). They are a source of bioactive substances, e.g., anthocyanidins and flavonols, and dietary fiber (2.4 g/100 g) [11]. Different parts of apples are characterized by different flavonoid concentrations, and it was shown that apple skin is the most abundant in them [12]. As apple peels are a rich source of antioxidative compounds and fiber, they could potentially act as a functional food in MetS prevention. On the other hand, industrial production of apple-derived products is often based on peeled fruits, which leads to a large amount of apple peel as industrial waste.

The main antioxidants present in apple peels are phenolic compounds [13]. However, the phenolic content and concentration could differ between apple varieties [14]. Idared and Rome Beauty varieties presented higher total phenolic content compared to other varieties, such as Cortland and Golden Delicious [13]. In detail, these compounds were flavonoids and anthocyanins. The content of flavonoids was similarly higher in peels of Rome Beauty, followed by Idared, then Cortland, and Golden Delicious at the end. For anthocyanins, the content was highest in Idared, then Cortland, Rome Beauty, and the lowest in Golden Delicious [13]. The analysis of the phenolic composition of the extract from Jonagold apple peels indicated that it is mainly composed of quercetin, catechin and epicatechin, anthocyanins such as cyanidin-3-O-galactoside, phloridzin, phloretin, and chlorogenic acid [15,16]. Similarly, Cripps Pink apple peels were also rich in quercetin, epicatechin, phloridzin, chlorogenic acid and anthocyanins [17].

Single compounds present in apple peels are suggested to potentially impact metabolic parameters related to MetS, such as lipid profile, blood pressure, glucose level, or fat mass [1,18,19,20,21].

Quercetin, which is the main flavonol. Its structure consists of pentahydroxyflavone, having the five hydroxy groups placed at the 3-, 3′-, 4′-, 5- and 7-positions. Its antioxidant activity is mainly dependent on its effect on glutathione level, enzymatic activity (mainly on acetylcholinesterase and butyrylcholinesterase), signal transduction pathways, and reactive oxygen species scavenging [22]. Quercetin is suggested to present cardioprotective properties, and it is found almost exclusively in apple peels [11,23]. It was shown that dietary quercetin intake was inversely correlated with BMI, waist circumference, and fat mass in obese patients; thus, it can suggest its potential in obesity prevention [20]. It was also shown that quercetin supplementation lowered blood pressure, and elevated blood pressure is one of the components of MetS [23]. Quercetin supplementation also resulted in lipid profile improvement, e.g., total cholesterol (TC) and low-density lipoprotein cholesterol (LDL) levels reduction, especially in smokers [24]. It also reduced the level of oxidized LDL, which is another well-known risk factor for atherosclerosis [25].

Cyanidin, another important antioxidative agent, was present only in red apple peels [11,23]. It consists of a flavylium cation backbone hydroxylated in different positions (generally on carbons C3, C5, C6, and C7 and C3′, C4′, and C5′) [26]. Its stability is highly dependent on pH, and its antioxidative role is based on reactive oxygen species scavenging. This way, it is suggested to reduce inflammation related to obesity [27]. The impact of purified anthocyanins supplementation on lipid profile was not significant [28]; however, the juices containing mainly cyanidin among other phenolic compounds, e.g., plum or red orange juice, were shown to reduce blood pressure, glucose, and LDL levels [29,30].

Moreover, apple peels are also a good source of dietary fiber [31]. Fiber is defined as a non-digestible form of carbohydrate plus lignin [32]. It impacts dietary carbohydrate absorption and also regulates the gut microbiome to produce less butyrate, which can be considered an indirect anti-inflammatory activity [32]. According to the meta-analysis by Chen et al., fiber consumption was inversely associated with general MetS risk in humans [33]. Regarding the components of MetS, there is a highly proven inverse relationship between fiber consumption and obesity in terms of body mass, waist circumference, and BMI [34,35,36]. It also decreases insulin resistance and diabetes type 2 risk [37], and this relationship was also proven in interventional human studies as arabinoxylan consumption decreased fasting glucose levels and insulin resistance parameters in patients with the prediabetic condition [38]. What is more, the positive role of fiber consumption in dyslipidemia prevention was proven in multiple observational human studies [39,40]. This impact was characterized mainly by HDL level increase and TC decrease [39]. This relationship was also confirmed in further interventional human studies [41,42]. According to the results of the Nutri-Net Sante study, hypertension could also be diminished by fiber consumption [43]. Blood pressure reduction after fiber implementation was also proven in meta-analyses by Streppel et al. and by Whelton et al. as well [44,45].

As presented above, the role of supplementation of single compounds present in apple peels could be potentially positive, but the results are not always consistent in human studies. On the other hand, the studies involving intervention with particular food products, e.g., juices, suggest such potential. This might be the result of the cooperation between single compounds as they could possibly modify each other functions. It was shown that antioxidative compounds present in apple peels could prevent from oxidation of other dietary components involved in cardiovascular prevention, such as fish oil, and interact with the gut microbiome [46,47]. Dietary fiber can also impact other aspects of dietary intake, such as energy dilution, appetite decrease, or, as already mentioned, altering the gut microbiome [48,49,50].

Moreover, when patients are advised to modify their dietary patterns, they consume particular products as a set of bioactive compounds. Thus, a potential dietary recommendation for real-life implementation should also focus on the role of particular products, not only separate chemical compounds. There is limited information available regarding the role of apple skin consumption or supplementation on metabolic parameters. The aim of this paper was to analyze the available literature regarding the impact of apple peel supplementation on metabolic syndrome components to summarize the foregoing findings and to suggest the direction of future research. The available papers have been devoted to the general apple consumption impact, while this review is the first one which focused on the potential of apple peels.

## 2. Materials and Methods

PubMed and Web of Science databases were searched for studies with the “apple peel” AND “metabolic” OR “lipid” OR “hypertension” OR “blood pressure” OR “glucose” OR “diabetes” OR “obesity” published until 21 January 2023. Only 1 human study was available, so the in vitro and animal studies were also included in this review.

## 3. Results

### 3.1. Obesity

Obesity is the foundation of MetS development. It is the main criterion that must be fulfilled to further diagnose MetS [1]. According to the International Obesity Task Force, obesity is defined as a BMI exceeding the 95th percentile [3]. The WHO European regional obesity report 2022 indicates that almost 60% of adults and 30% of school children in Europe are affected by obesity [51]. Worldwide the number of obesity-affected individuals has tripled since 1975. People with obesity have been found to have 30% higher treatment costs than those without, while there may also be indirect costs due to earnings lost as a result of premature mortality and obesity-related disability [52]. As described above, excessive fat tissue, which is the main obesity characteristic, leads to low-grade chronic inflammation and adipokine production [2]. They result in endothelial and metabolic abnormalities, which then consequence in clinical manifestations of MetS components, i.e., elevated blood pressure, atherogenic dyslipidemia, and elevated glucose level [1]. However, obesity and accompanying inflammation are the basis of MetS development.

In vitro studies suggested the anti-obesity potential of apple peel extract, as it reduced the level of lipid accumulation by pre-adipocytes [53].

Elkahoui et al. designed an animal experiment with MetS induced by a high-fat diet, evaluating apple peel supplementation’s effect on standard metabolic parameters. There was observed a dose-response impact on weight gain reduction and adipose tissue mass reduction in high-fat-fed mice; however, this relationship was not significant [54]. Similarly, it did not prevent general obesity development in high-fat-fed mice; however, it led to other positive metabolic changes, which are described below [55]. The authors of the study also indicated the important role of unabsorbed dietary components on gut microbiota, altering it even more compared to concentrated high-phenolic apple extract. The results were considered intriguing, as the microbiome is a complex issue related to many compounds and needs extensive further studies, but the valorization of this food-processing waste product was concluded with apparent health effects on the obesity-related state of the organism.

The details of the mentioned studies are presented in Table 1. Unfortunately, there are no available observations in humans on this topic.

### 3.2. Lipid Profile

Atherogenic dyslipidemia is a condition defined as elevated TG and LDL levels and reduced HDL levels [61]. This configuration is a recognized factor of atherosclerosis and the development of its clinical complications, such as coronary artery disease and ischemic stroke [62]. The MetS criteria include a TG level of 150 mg/dL (1.7 mmol/L) or higher, or specific treatment for this lipid abnormality, and an HDL level lower than 40 mg/dL (1.03 mmol/L) in males or lower than 50 mg/dL (1.29 mmol/L) in females, or specific treatment for this lipid abnormality [1].

The study by Gonzalez et al. showed that a diet supplemented in apple-peel reduced the lipid parameters in the MetS mice model [56]. It is worth noting that the reduction was observed for all: TC, HDL, LDL, and TG [56]. The more desired changes were observed in both: high-choline fed and high-fructose fed mice as apple peel supplementation led to HDL increase and TC, TG and LDL decrease [12,57]. Similar observations were made in hyperlipidemic rats, as apple peel supplementation significantly reduced serum LDL and increased HDL levels [58] and in diabetic rats, where a decrease in TC, LDL and TG with significant HDL elevation was observed [59]. This relationship was also present in tilapia, as apple peel powder supplementation reduced TC and TG levels in both the serum and liver [60].

On the other hand, atherosclerosis development is also dependent on antioxidative status. Oxidized LDL is known to lead to faster development and progression than normal LDL. That is why it is worth noting that apart from the positive impact on lipid profile, apple peel supplementation lead also to higher plasma antioxidant capacity in cholesterol-fed rats [63]. These observations are consistent with the results of the in vitro studies in human cells, as metabolites from apple peel extract inhibited human LDL oxidation [15]. These results were mainly caused by quercetin metabolites. In diabetic rats, apple peel supplementation leads to the reduction of tumor necrosis factor α, interleukin 6, and interleukin 8 levels, which are inflammation mediators [59].

There is only one small human study that analyzed the lipid profile in the context of apple peel consumption. It showed that daily consumption of two apples with skin did not change LDL, HDL, TC, or TG levels. These differences might be caused by the variances in applied phenolic doses. It was calculated that human participants consumed approximately 306 mg/day of total phenolic compounds, while there were relatively higher doses used in the presented animal model studies, i.e., from 114 mg/kg to 1 g/kg [58,59]. The presented human study was also based on the small inhomogeneous metabolically group, so more studies in selected larger human groups could clarify this issue. The details of the mentioned studies are presented in Table 1.

### 3.3. Glucose Level

Excessive fat tissue is a source of adipokines, such as resistin, which leads to insulin resistance [64]. Secondarily, it results in elevated blood glucose levels, which can be classified as prediabetes or diabetes, depending on the level of present disturbances [65]. Diabetes is an important cardiovascular risk factor; thus, proper glycemia management is crucial in terms of cardiovascular prevention [66]. The cut-off for the MetS criterion is a fasting glucose level of 100 mg/dL (5.6 mmol/L) or higher (which is lower than for diabetes diagnosis) or previously diagnosed type 2 diabetes [1].

Apple peel supplementation diets reduced glycemia in MetS mice [12,56]. What is more, insulin level reduction was also observed, which could suggest insulin resistance reduction [56]. Moreover, in animal models, apple peel supplementation led to significantly lower glucose levels in high-fat-fed obese mice [55]. These properties were also observed in diabetic rats, where apple peel supplementation reduced fasting glucose levels [59]. On the other hand, these properties were not confirmed in the only one available, already mentioned, a human study that analyzed this parameter [17]. It might have been caused by the already pointed out methodological aspects of the presented study.

### 3.4. Blood Pressure

Elevated blood pressure is another component of MetS. The cut-off for the MetS criterion is a systolic blood pressure level of 130 or higher, a diastolic blood pressure level of 85 mm Hg or higher (which is lower than hypertension diagnosis), or treatment of previously diagnosed hypertension [1]. It was shown that high values of systolic blood pressure correspond with higher cardiovascular risk [66]. The dietary approach against hypertension is a nutritional pattern that promotes high fruit and vegetable intake [67]. It was proven as a successful behavioral intervention for hypertension management and is recommended by the European Society of Cardiology [68].

The blood pressure level is the result of the balance between vasoconstrictors and vasodilators produced by endothelium. Nitric oxide is a recognized vasorelaxant, while endothelin-1 is one of the main vasoconstrictors. Apple peel supplementation led to nitric oxide level elevation and endothelin-1 level decrease in high choline-fed and high-fructose-fed mice [12,57]. The effects were higher for apple peel compared to apple flesh [12]. This could suggest a potential for blood pressure reduction in the course of apple peel supplementation. On the other hand, daily consumption of two apples with skin did not significantly change the nitric oxide level or 24 h blood pressure level in humans, not acutely, nor after 4 weeks [17]. As already described, these inconsistencies might be caused by the differences in phenolic doses and inclusion criteria.

Angiotensin is another agent responsible for blood pressure level elevation. It is produced with the angiotensin-converting enzyme (ACE). Apple peel extract inhibited ACE [16]. It is worth noting that flavonoids derived from apple peel are competitive inhibitors of ACE, and quercetin metabolites turned out to be the strongest ones [16].

There is only one human study that investigated the impact of apples on skin consumption of endothelial function and showed a significant increase in flow-mediated dilation of the brachial artery acutely and after four weeks of chronic intake compared to peeled apple consumption [17]. This may still suggest a subtle potential role of apple peel supplementation in blood pressure management.

## 4. Discussion

MetS is associated with modern societies’ lifestyle changes, such as diet modification and lack of physical activity. It has become a global healthcare problem. The estimated prevalence of MetS in Europe was about 24% in 2015, while it was even over 35% in the United States of America in 2012 [69,70]. MetS is not an isolated disease (it does not have a separate International Classification of Diseases ICD-10 code), but it is defined as a set of metabolic disorders primarily caused by central obesity [71]. As already mentioned, they include insulin resistance, atherogenic dyslipidemia, central obesity, and elevated blood pressure. Due to the fact that MetS is a cluster of multiple diseases or disorders, it has a very high potential of impacting cardiovascular risk, as each of its components separately is a cardiovascular risk factor itself. This aspect is very important as cardiovascular diseases and diabetes, which are also a part of MetS, are the leading causes of death globally [72].

The MetS pathogenesis is complex. It primarily initiates from central obesity. Excessive adipose tissue location is an important factor as visceral adipose tissue is more vulnerable to macrophage infiltration and then inflammation development and free fatty acids release [73,74]. This way, excessive fat tissue located in the abdomen area is more active in terms of adipokines production. Adiponectin is one of them and impacts lipid and glucose metabolism, which further results in free fatty acid and inflammatory cytokine production [75,76]. This way, it leads to insulin resistance, chronic low-grade general inflammation, and neurohormonal activation. These processes then lead to endothelium dysfunction and glucose and lipid metabolism disorders. To sum up, obesity underlying MetS leads to low-grade systemic inflammation, which then results in multiple metabolic disorders. All of them are cardiovascular risk factors, which is why MetS prevention and treatment is one of the main healthcare problems.

Apple peels are a source of antioxidative agents, e.g., quercetin and cyanidin, and other important nutrients, such as dietary fiber [31,77]. As already mentioned, a positive relationship between quercetin supplementation and MetS-related factors was reported with different significance, indicating some potentially beneficial changes in the literature. They were observed mainly for obesity and blood pressure reduction. Similarly, cyanidin alone was also suggested to potentially improve MetS components; however, the results are not consistent.

Apple peels are characterized by a higher concentration of antioxidative agents compared to apple flesh; however, on the other hand, it is worth noting that apple peel can also contain more pesticides residues, e.g., chlorpyrifos, β-cypermethrin, tebuconazole, acetamiprid or carbendazim [78,79]. That is why it is important to use ecological sources of apples and wash the fruit carefully when peels are planned to be consumed or supplemented.

It is worth mentioning that apple peels are a rich source of fiber, which is indirectly connected with MetS [31]. It is not an active compound modifying pathways, but it rather regulates metabolism, e.g., by slowing down the absorption of carbohydrates, thereby reducing the risk for patients with type 2 diabetes and prediabetic conditions by avoiding high blood sugar levels [80]. Fiber can promote a feeling of fullness, leading to lower caloric intake, especially in overweight and obese patients [48,49]. The higher fiber content in diets also helps to remove excess salt and water, resulting in lower blood pressure [49]. It is worth indicating that the abovementioned modulation of intake lowers cholesterol levels, translating to lower cardiovascular risk in patients. Some of the results referenced the gut microbiota as an important factor in the health and pathology of various diseases. Overall health can be improved by maintaining a healthy gut microbiome related to a high-fiber diet, as according to Benitez–Peaez et al., consumption of a balanced diet reduces inflammation and protects against harmful microorganisms [49,50].

In vitro and animal model studies suggest the positive role of apple peel supplementation in terms of each of the MetS components, i.e., obesity, elevated blood pressure, atherogenic dyslipidemia, and elevated glucose level as the result of insulin resistance. Apple peel supplementation resulted in body mass and fat mass reduction, but these relationships were not significant. However, when obesity was already present, apple peel supplementation led to a diminishment of other metabolic disorders which altogether are the components of MetS. Dyslipidemia is one of the components of MetS. It covers two of the MetS criteria, i.e., elevated TG level and reduced HDL level. Animal model studies showed that apple peel supplementation resulted in positive changes in lipid profile, such as TG level reduction and HDL level increase. LDL elevation is a lipid disorder also present in MetS but not directly captured in MetS criteria, similar to LDL oxidation. Animal model studies showed a significant reduction of LDL levels after apple peel supplantation. Moreover, in vitro studies proved that apple peel supplementation results in LDL oxidation inhibition. It is worth noting that all of the described above lipid disorders lead to atherosclerosis development, which is why they are recognized cardiovascular risk factors.

Adipokine production by the excessive visceral adipose tissue leads to insulin resistance. The clinical effect of this phenomenon is elevated glucose levels. Initially, it refers only to particular situations, and it is defined as prediabetes, i.e., impaired fasting glucose or impaired glucose tolerance, but then it progresses into diabetes type 2. Elevated glucose level leads to multiple complications such as micro- and macroangiopathies, including atherosclerosis promotion and neuropathies development. This way, prediabetes and diabetes are also cardiovascular risk factors. Animal studies showed that apple peel supplementation leads to the improvement of the major disorders which are the foundation of diabetes, i.e., insulin and fasting glucose levels decrease.

**Figure 1:**
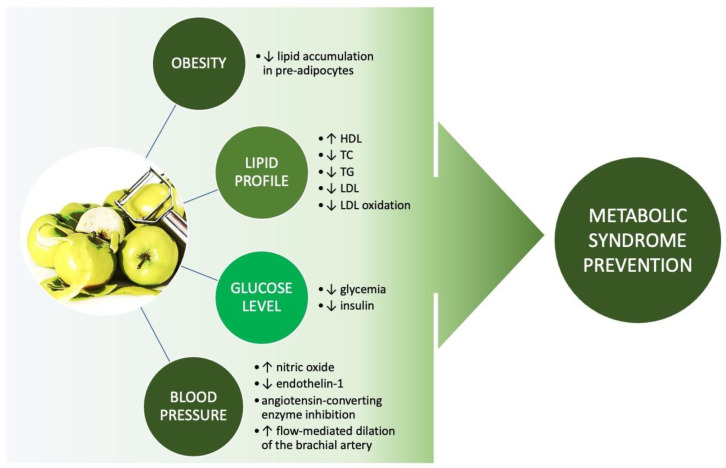
The impact of apple peel supplementation on the MetS components (TC—total cholesterol, TG—triglycerides, HDL—high-density lipoprotein cholesterol, LDL—low-density lipoprotein cholesterol, ↓—decrease, ↑—increase).

Central obesity can also conduce to elevated pressure development. It can be the result of elevated body mass or of the described above bioactivity of excessive fat tissue. Altogether they result in the disruption of blood pressure regulation mechanisms. In vitro studies suggest the possibility that apple peel extract can inhibit ACE, which is one of the main enzymes involved in blood pressure regulation. Animal model studies also suggest other potential pathways, such as nitric oxide level elevation and endothelin-1 level reduction. All of these effects of apple peel supplementation should potentially clinically lead to a blood pressure level decrease. On the other hand, there is only one human study available on this complex topic. It showed that apple peel supplementation had an impact only on indirect parameters related to blood pressure, i.e., flow-mediated dilation of the brachial artery, while the other clinical parameters important such as mean 24 h blood pressure level, fasting glucose level, lipid profile in MetS remained unaltered. As described above, the inconsistency between animal model studies and this human study might be the result of methodological differences. These differences refer to the used doses of apple peel or apple peel extract, as it was proportionally higher in animal model studies compared to the mentioned human study. The important fact is also that this study compared the consumption of apples with skin to apple flesh, not to placebo. Another important aspect referred to the study group. In vitro studies are performed in highly controlled conditions. Similarly, the animal model requires induction of MetS in selected animals and enables full control of each aspect of the study group; thus, it involved a very homogenous study group. On the contrary, the presented human study included the participants with one of any of the MetS criteria: elevated blood pressure or elevated glucose level or central obesity or elevated total cholesterol (which is not directly captured in the MetS criteria). As already described above, MetS diagnosis requires fulfilling three of MetS criteria (obesity plus two additional criteria), so this study did not include properly MetS-diagnosed patients. This fact made the study group less metabolically homogenous. Moreover, it also involved a relatively small group of participants, i.e., 30 people, so it should be treated more like a pilot study. All of these aspects should be taken into consideration, and that is why more human studies are needed to clarify the potential role of apple peel supplementation in MetS. The summarized results and conclusion of this review are presented in Figure 1.

## 5. Conclusions

The results of available in vitro and animal model studies suggest a positive role of apple peel supplementation in the MetS components’ management. There is only one human study available that does not clinically confirm these results; however, methodological aspects should be considered. More observations from larger and homogenous metabolically human groups are needed to clarify the role of apple peel supplementation in MetS management.

### Limitations of the Study

This review covers the narrow topic referring to nutrition with a focus on the particular food product and MetS. The number of available studies was limited, so the results should be interpreted with great caution. Also, there was only one human study available to discuss, so more human studies are needed to verify the suggested relationships.

## Figures and Tables

**Table 1:** The details of the studies focused on apple peel supplementation’s impact on metabolic syndrome components.

Reference	Material	Form and Daily Dose	Effects
[53]	pre-adipocytes	apple peel extract; 1 μg dry powder/1 mL	↓ lipid accumulation
[54]	high-fat fed mice	apple peel powder; 100 and 200 g/kg diet	non-significant ↓ weight gain and ↓ adipose tissue mass
[56]	metabolic syndrome mice	unknown form; 200 g/kg diet	↓ TC, ↓ HDL, ↓ LDL, ↓ TG, ↓ glycemia, ↓ insulin
[57]	high-choline fed	apple peel extract; 600 and 900 mg/kg body weight	↑ HDL and ↓ TC, ↓ TG, ↓ LDL, ↑ nitric oxide, ↓ endothelin-1
[12]	high-fat and high-fructose-fed mice	apple peel extract; 250 mg/kg	↑ HDL, ↓ TC, ↓ TG, ↓ LDL, ↑ nitric oxide, ↓ endothelin-1, ↓ glycemia
[58]	hyperlipidemic rats	apple peel extract; 57 and 114 mg/kg body weight	↓ LDL, ↑ HDL
[59]	diabetic rats	apple peel extract; 1 g/kg	↓ TC, ↓ LDL, ↓ TG, ↑ HDL, ↓ glycemia
[60]	tilapia	apple peel powder; 0.2% of diet	↓ TC, ↓ TG
[15]	human cells	quercetin rich apple peel extract; 10 mg/L	↓ LDL oxidation
[55]	high-fat fed mice	apple peel extract; 200 g/kg diet	↓ glycemia
[17]	humans with mixed metabolic disorders	Cripps Pink apple peel; 33.8 g	↑ flow-mediated dilation of the brachial artery; no changes in lipid profile, glycemia, nitric oxide or 24 h blood pressure level
[16]	in vitro	apple peel extract; 49 mg/L	angiotensin-converting enzyme inhibition

TC—total cholesterol, TG—triglycerides, HDL—high-density lipoprotein cholesterol, LDL—low-density lipoprotein cholesterol, ↓—decrease, ↑—increase.

## Data Availability

The data that support the findings of this study are available from the corresponding author upon reasonable request.

## References

[1] (2005). International Diabetes Federation The IDF consensus worldwide definition of the metabolic syndrome. Obes. Metab..

[2] Kawai T., Autieri M.V., Scalia R. (2021). Adipose tissue inflammation and metabolic dysfunction in obesity. Am. J. Physiol. Physiol..

[3] Alberti K.G.M.M., Eckel R.H., Grundy S.M., Zimmet P.Z., Cleeman J.I., Donato K.A., Fruchart J.C., James W.P.T., Loria C.M., Smith S.C. (2009). Harmonizing the Metabolic Syndrome: A Joint Interim Statement of the International Diabetes Federation Task Force on Epidemiology and Prevention; National Heart, Lung, and Blood Institute; American Heart Association; World Heart Federation; International Atherosclerosis Society; And International Association for the Study of Obesity. Circulation.

[4] Eckel R.H., Alberti K.G.M.M., Grundy S.M., Zimmet P.Z. (2010). The metabolic syndrome. Lancet.

[5] Alshammary A.F., Alharbi K.K., Alshehri N.J., Vennu V., Khan I.A. (2021). Metabolic Syndrome and Coronary Artery Disease Risk: A Meta-Analysis of Observational Studies. Int. J. Environ. Res. Public Health.

[6] Raposo L. (2021). Metabolic syndrome in Poland: The WOBASZ II study. Pol. Arch. Intern. Med..

[7] Popiolek-Kalisz J. (2023). The Relationship between Dietary Flavonols Intake and Metabolic Syndrome in Polish Adults. Nutrients.

[8] World Health Organization (WHO) (2000). Obesity: Preventing and Managing the Global Epidemic. Report of a WHO Consultation. World Health Organ. Tech. Rep. Ser..

[9] Gallardo-Alfaro L., Bibiloni M.D.M., Mascaró C.M., Montemayor S., Ruiz-Canela M., Salas-Salvadó J., Corella D., Fitó M., Romaguera D., Vioque J. (2020). Leisure-Time Physical Activity, Sedentary Behaviour and Diet Quality are Associated with Metabolic Syndrome Severity: The PREDIMED-Plus Study. Nutrients.

[10] Shin J.Y., Kim J.Y., Kang H.T., Han K.H., Shim J.Y. (2015). Effect of fruits and vegetables on metabolic syndrome: A systematic review and meta-analysis of randomized controlled trials. Int. J. Food Sci. Nutr..

[11] Tsao R., Yang R., Young J.C., Zhu H. (2003). Polyphenolic Profiles in Eight Apple Cultivars Using High-Performance Liquid Chromatography (HPLC). J. Agric. Food Chem..

[12] Tian J., Wu X., Zhang M., Zhou Z., Liu Y. (2017). Comparative study on the effects of apple peel polyphenols and apple flesh polyphenols on cardiovascular risk factors in mice. Clin. Exp. Hypertens..

[13] Wolfe K., Wu X., Liu R.H. (2003). Antioxidant Activity of Apple Peels. J. Agric. Food Chem..

[14] Kalinowska M., Gryko K., Wróblewska A.M., Jabłońska-Trypuć A., Karpowicz D. (2020). Phenolic content, chemical composition and anti-/pro-oxidant activity of Gold Milenium and Papierowka apple peel extracts. Sci. Rep..

[15] Thilakarathna S.H., Rupasinghe H.V., Needs P.W. (2013). Apple peel bioactive rich extracts effectively inhibit in vitro human LDL cholesterol oxidation. Food Chem..

[16] Balasuriya N., Rupasinghe H.V. (2012). Antihypertensive properties of flavonoid-rich apple peel extract. Food Chem..

[17] Bondonno N., Bondonno C.P., Blekkenhorst L., Considine M., Maghzal G., Stocker R., Woodman R., Ward N., Hodgson J.M., Croft K. (2018). Flavonoid-Rich Apple Improves Endothelial Function in Individuals at Risk for Cardiovascular Disease: A Randomized Controlled Clinical Trial. Mol. Nutr. Food Res..

[18] Ostadmohammadi V., Milajerdi A., Ayati E., Kolahdooz F., Asemi Z. (2019). Effects of quercetin supplementation on glycemic control among patients with metabolic syndrome and related disorders: A systematic review and meta-analysis of randomized controlled trials. Phytotherapy Res..

[19] Popiolek-Kalisz J., Blaszczak P., Fornal E. (2022). Dietary Isorhamnetin Intake Is Associated with Lower Blood Pressure in Coronary Artery Disease Patients. Nutrients.

[20] Popiolek-Kalisz J. (2022). The Impact of Dietary Flavonols on Central Obesity Parameters in Polish Adults. Nutrients.

[21] Popiolek-Kalisz J., Fornal E. (2022). Dietary Isorhamnetin Intake Is Inversely Associated with Coronary Artery Disease Occurrence in Polish Adults. Int. J. Environ. Res. Public Health.

[22] Xu D., Hu M.-J., Wang Y.-Q., Cui Y.-L. (2019). Antioxidant Activities of Quercetin and Its Complexes for Medicinal Application. Molecules.

[23] Popiolek-Kalisz J., Fornal E. (2022). The Effects of Quercetin Supplementation on Blood Pressure–Meta-Analysis. Curr. Probl. Cardiol..

[24] Lee K.-H., Park E., Lee H.-J., Kim M.-O., Cha Y.-J., Kim J.-M., Lee H., Shin M.-J. (2011). Effects of daily quercetin-rich supplementation on cardiometabolic risks in male smokers. Nutr. Res. Pract..

[25] Egert S., Bosy-Westphal A., Seiberl J., Kürbitz C., Settler U., Plachta-Danielzik S., Wagner A.E., Frank J., Schrezenmeir J., Rimbach G. (2009). Quercetin reduces systolic blood pressure and plasma oxidised low-density lipoprotein concentrations in overweight subjects with a high-cardiovascular disease risk phenotype: A double-blinded, placebo-controlled cross-over study. Br. J. Nutr..

[26] Mattioli R., Francioso A., Mosca L., Silva P. (2020). Anthocyanins: A Comprehensive Review of Their Chemical Properties and Health Effects on Cardiovascular and Neurodegenerative Diseases. Molecules.

[27] Lee Y.-M., Yoon Y., Yoon H., Park H.-M., Song S., Yeum K.-J. (2017). Dietary Anthocyanins against Obesity and Inflammation. Nutrients.

[28] Vugic L., Colson N., Nikbakht E., Gaiz A., Holland O.J., Kundur A.R., Singh I. (2020). Anthocyanin supplementation inhibits secretion of pro-inflammatory cytokines in overweight and obese individuals. J. Funct. Foods.

[29] Bhaswant M., Brown L., Mathai M.L. (2019). Queen Garnet plum juice and raspberry cordial in mildly hypertensive obese or overweight subjects: A randomized, double-blind study. J. Funct. Foods.

[30] Azzini E., Venneria E., Ciarapica D., Foddai M.S., Intorre F., Zaccaria M., Maiani F., Palomba L., Barnaba L., Tubili C. (2017). Effect of Red Orange Juice Consumption on Body Composition and Nutritional Status in Overweight/Obese Female: A Pilot Study. Oxidative Med. Cell. Longev..

[31] Henríquez C., Speisky H., Chiffelle I., Valenzuela T., Araya M., Simpson R., Almonacid S. (2010). Development of an Ingredient Containing Apple Peel, as a Source of Polyphenols and Dietary Fiber. J. Food Sci..

[32] Barber T., Kabisch S., Pfeiffer A., Weickert M. (2020). The Health Benefits of Dietary Fibre. Nutrients.

[33] Chen J.-P., Chen G.-C., Wang X.-P., Qin L., Bai Y. (2018). Dietary Fiber and Metabolic Syndrome: A Meta-Analysis and Review of Related Mechanisms. Nutrients.

[34] Du H., van der A D.L., Boshuizen H.C., Forouhi N.G., Wareham N.J., Halkjær J., Tjønneland A., Overvad K., Jakobsen M.U., Boeing H. (2010). Dietary fiber and subsequent changes in body weight and waist circumference in European men and women. Am. J. Clin. Nutr..

[35] Davis J.N., Alexander K.E., Ventura E.E., Toledo-Corral C.M., Goran M.I. (2009). Inverse relation between dietary fiber intake and visceral adiposity in overweight Latino youth. Am. J. Clin. Nutr..

[36] Kromhout D., Bloemberg B., Seidell J., Nissinen A., Menotti A., for the Seven Countries Study Group (2001). Physical activity and dietary fiber determine population body fat levels: The Seven Countries Study. Int. J. Obes..

[37] Cho S.S., Qi L., Fahey G.C., Klurfeld D. (2013). Consumption of cereal fiber, mixtures of whole grains and bran, and whole grains and risk reduction in type 2 diabetes, obesity, and cardiovascular disease. Am. J. Clin. Nutr..

[38] Garcia A.L., Otto B., Reich S.-C., Weickert M.O., Steiniger J., Machowetz A., Rudovich N.N., Möhlig M., Katz N., Speth M. (2007). Arabinoxylan consumption decreases postprandial serum glucose, serum insulin and plasma total ghrelin response in subjects with impaired glucose tolerance. Eur. J. Clin. Nutr..

[39] Zhou Q., Wu J., Tang J., Wang J.-J., Lu C.-H., Wang P.-X. (2015). Beneficial Effect of Higher Dietary Fiber Intake on Plasma HDL-C and TC/HDL-C Ratio among Chinese Rural-to-Urban Migrant Workers. Int. J. Environ. Res. Public Health.

[40] Shinozaki K., Okuda M., Kunitsugu I., Shigeta M., Sasaki S. (2015). Dietary Fiber Consumption Decreases the Risks of Overweight and Hypercholesterolemia in Japanese Children. Ann. Nutr. Metab..

[41] Hollænder P.L., Ross A.B., Kristensen M. (2015). Whole-grain and blood lipid changes in apparently healthy adults: A systematic review and meta-analysis of randomized controlled studies. Am. J. Clin. Nutr..

[42] Zhu X., Sun X., Wang M., Zhang C., Cao Y., Mo G., Liang J., Zhu S. (2015). Quantitative assessment of the effects of beta-glucan consumption on serum lipid profile and glucose level in hypercholesterolemic subjects. Nutr. Metab. Cardiovasc. Dis..

[43] Lelong H., Blacher J., Baudry J., Adriouch S., Galan P., Fezeu L., Hercberg S., Kesse-Guyot E., Günther A.L., Liese A.D. (2017). Individual and Combined Effects of Dietary Factors on Risk of Incident Hypertension Prospective Analysis from the Nutrinet-Santé Cohort. Hypertension.

[44] Streppel M.T., Arends L.R., Van’t Veer P., Grobbee D.E., Geleijnse J.M. (2005). Dietary Fiber and Blood Pressure: A Meta-Analysis of Randomized Placebo-Controlled Trials. Arch. Intern. Med..

[45] Whelton S.P., Hyre A.D., Pedersen B., Yi Y., Whelton P.K., He J. (2005). Effect of dietary fiber intake on blood pressure: A meta-analysis of randomized, controlled clinical trials. J. Hypertens..

[46] Sekhon-Loodu S., Warnakulasuriya S.N., Rupasinghe H.V., Shahidi F. (2013). Antioxidant ability of fractionated apple peel phenolics to inhibit fish oil oxidation. Food Chem..

[47] Gil Cardoso K., Ginés I., Pinent M., Ardévol A., Blay M.T., Terra X. (2016). Effects of flavonoids on intestinal inflammation, barrier integrity and changes in gut microbiota during diet-induced obesity. Nutr. Res. Rev..

[48] Howarth N.C., Saltzman E., Roberts S.B. (2001). Dietary Fiber and Weight Regulation. Nutr. Rev..

[49] Lee I., Shi L., Webb D.-L., Hellström P.M., Risérus U., Landberg R. (2016). Effects of whole-grain rye porridge with added inulin and wheat gluten on appetite, gut fermentation and postprandial glucose metabolism: A randomised, cross-over, breakfast study. Br. J. Nutr..

[50] Benítez-Páez A., Del Pulgar E.M.G., Kjølbæk L., Brahe L.K., Astrup A., Larsen L., Sanz Y. (2016). Impact of dietary fiber and fat on gut microbiota re-modeling and metabolic health. Trends Food Sci. Technol..

[51] World Health Organization (2022). WHO European Regional Obesity Report 2022.

[52] Withrow D., Alter D.A. (2011). The economic burden of obesity worldwide: A systematic review of the direct costs of obesity. Obes. Rev..

[53] Ko D.-Y., Ku K.-M. (2022). Effect of Anti-Obesity and Antioxidant Activity through the Additional Consumption of Peel from ‘Fuji’ Pre-Washed Apple. Foods.

[54] Elkahoui S., Levin C.E., Bartley G.E., Yokoyama W., Friedman M. (2019). Levels of Fecal Procyanidins and Changes in Microbiota and Metabolism in Mice Fed a High-Fat Diet Supplemented with Apple Peel. J. Agric. Food Chem..

[55] Snyder S.M., Zhao B., Luo T., Kaiser C., Cavender G., Hamilton-Reeves J., Sullivan D.K., Shay N.F. (2016). Consumption of Quercetin and Quercetin-Containing Apple and Cherry Extracts Affects Blood Glucose Concentration, Hepatic Metabolism, and Gene Expression Patterns in Obese C57BL/6J High Fat–Fed Mice. J. Nutr..

[56] Gonzalez J., Donoso W., Sandoval N., Reyes M., Gonzalez P., Gajardo M., Morales E., Neira A., Razmilic I., Yuri J.A. (2015). Apple Peel Supplemented Diet Reduces Parameters of Metabolic Syndrome and Atherogenic Progression in ApoE−/− Mice. Evid.-Based Complement. Altern. Med..

[57] Jia M., Ren D., Nie Y., Yang X. (2017). Beneficial effects of apple peel polyphenols on vascular endothelial dysfunction and liver injury in high choline-fed mice. Food Funct..

[58] Susilowati R., Jannah J., Maghfuroh Z., Kusuma M.T. (2020). Antihyperlipidemic effects of apple peel extract in high-fat diet-induced hyperlipidemic rats. J. Adv. Pharm. Technol. Res..

[59] Fathy S.M., Drees E.A. (2015). Protective effects of Egyptian cloudy apple juice and apple peel extract on lipid peroxidation, antioxidant enzymes and inflammatory status in diabetic rat pancreas. BMC Complement. Altern. Med..

[60] Qiang J., Khamis O.A.M., Jiang H.J., Cao Z.M., He J., Tao Y.F., Xu P., Bao J.W. (2019). Effects of dietary supplementation with apple peel powder on the growth, blood and liver parameters, and transcriptome of genetically improved farmed tilapia (GIFT, Oreochromis niloticus). PLoS ONE.

[61] Mach F., Baigent C., Catapano A.L., Koskinas K.C., Casula M., Badimon L., Chapman M.J., De Backer G.G., Delgado V., Ference B.A. (2020). 2019 ESC/EAS Guidelines for the Management of Dyslipidaemias: Lipid Modification to Reduce Cardiovascular Risk. Eur. Heart J..

[62] Knuuti J., Wijns W., Saraste A., Capodanno D., Barbato E., Funck-Brentano C., Prescott E., Storey R.F., Deaton C., Cuisset T. (2020). 2019 ESC Guidelines for the diagnosis and management of chronic coronary syndromes. Eur. Heart J..

[63] Leontowicz M., Gorinstein S., Leontowicz H., Krzeminski R., Lojek A., Katrich E., Číž M., Martin-Belloso O., Soliva-Fortuny R., Haruenkit R. (2003). Apple and Pear Peel and Pulp and Their Influence on Plasma Lipids and Antioxidant Potentials in Rats Fed Cholesterol-Containing Diets. J. Agric. Food Chem..

[64] Dzięgielewska-Gęsiak S., Wyszomirska K., Fatyga E., Wysocka E., Muc-Wierzgoń M. (2021). The role of oxidant-antioxidant markers and resistin in metabolic syndrome elderly individuals. Sci. Prog..

[65] American Diabetes Association 2 (2021). Classification and Diagnosis of Diabetes: Standards of Medical Care in Diabetes—2022. Diabetes Care.

[66] Visseren F.L.J., Mach F., Smulders Y.M., Carballo D., Koskinas K.C., Bäck M., Benetos A., Biffi A., Boavida J.-M., Capodanno D. (2021). ESC Guidelines on cardiovascular disease prevention in clinical practice. Eur. Heart J..

[67] Chiu S., Bergeron N., Williams P.T., A Bray G., Sutherland B., Krauss R.M. (2016). Comparison of the DASH (Dietary Approaches to Stop Hypertension) diet and a higher-fat DASH diet on blood pressure and lipids and lipoproteins: A randomized controlled trial. Am. J. Clin. Nutr..

[68] Williams B., Mancia G., Spiering W., Agabiti Rosei E., Azizi M., Burnier M., Clement D., Coca A., De Simone G., Dominiczak A. (2018). 2018 Practice guidelines for the management of arterial hypertension of the European Society of Cardiology and the European Society of Hypertension. Blood Press..

[69] Moore J.X., Chaudhary N., Akinyemiju T. (2017). Metabolic Syndrome Prevalence by Race/Ethnicity and Sex in the United States, National Health and Nutrition Examination Survey, 1988–2012. Prev. Chronic Dis..

[70] Scuteri A., Laurent S., Cucca F., Cockcroft J., Cunha P., Rodríguez-Mañas L., Raso F.U.M., Muiesan M.L., Ryliškytė L., Rietzschel E. (2014). Metabolic syndrome across Europe: Different clusters of risk factors. Eur. J. Prev. Cardiol..

[71] Matsuzawa Y., Funahashi T., Nakamura T. (2011). The Concept of Metabolic Syndrome: Contribution of Visceral Fat Accumulation and Its Molecular Mechanism. J. Atheroscler. Thromb..

[72] Roth G.A., Abate D., Abate K.H., Abay S.M., Abbafati C., Abbasi N., Abbastabar H., Abd-Allah F., Abdela J., Abdelalim A. (2018). Global, regional, and national age-sex-specific mortality for 282 causes of death in 195 countries and territories, 1980–2017: A systematic analysis for the Global Burden of Disease Study 2017; GBD 2017 Causes of Death Collaborators. Lancet.

[73] Patel P., Abate N. (2013). Body Fat Distribution and Insulin Resistance. Nutrients.

[74] Blaszczak A.M., Jalilvand A., Liu J., Wright V.P., Suzo A., Needleman B., Noria S., Lafuse W., Hsueh W.A., Bradley D. (2019). Human Visceral Adipose Tissue Macrophages Are Not Adequately Defined by Standard Methods of Characterization. J. Diabetes Res..

[75] Ghadge A.A., Khaire A.A., Kuvalekar A.A. (2018). Adiponectin: A potential therapeutic target for metabolic syndrome. Cytokine Growth Factor Rev..

[76] Kadowaki T., Yamauchi T., Kubota N., Hara K., Ueki K., Tobe K. (2006). Adiponectin and adiponectin receptors in insulin resistance, diabetes, and the metabolic syndrome. J. Clin. Investig..

[77] Wolfe K.L., Liu R.H. (2003). Apple Peels as a Value-Added Food Ingredient. J. Agric. Food Chem..

[78] Kong Z., Shan W., Dong F., Liu X., Xu J., Li M., Zheng Y. (2012). Effect of home processing on the distribution and reduction of pesticide residues in apples. Food Addit. Contam. Part A.

[79] Rasmusssen R.R., Poulsen M.E., Hansen H.C.B. (2003). Distribution of multiple pesticide residues in apple segments after home processing. Food Addit. Contam..

[80] Eastwood M.A., Morris E.R. (1992). Physical properties of dietary fiber that influence physiological function: A model for polymers along the gastrointestinal tract. Am. J. Clin. Nutr..

